# Determining the Effects of Compost Substitution on Carbon Sequestration, Greenhouse Gas Emission, Soil Microbial Community Changes, and Crop Yield in a Wheat Field

**DOI:** 10.3390/life12091382

**Published:** 2022-09-05

**Authors:** Hongzhi Min, Xingchen Huang, Daoqing Xu, Qingqin Shao, Qing Li, Hong Wang, Lantian Ren

**Affiliations:** 1 Engineering Research Center for Smart Crop Planting and Processing Technology, Anhui Science and Technology University, Chuzhou 233100, China; 2 Cotton Research Institute of Anhui Academy of Agricultural Sciences, Hefei 230036, China; 3 College of Plant Science & Technology, Hua Zhong Agricultural University, Wuhan 430070, China

**Keywords:** composting, wheat, yield, greenhouse gas, soil micro-organism

## Abstract

Compost produced by straw and livestock and poultry manure under the action of micro-organisms is one of the main forms of organic alternative fertilizers at present. The present study explored the effects of compost substitution on soil greenhouse gas emissions, soil microbial community changes, and wheat yield to determine the best substitution ratio for reducing greenhouse gas emissions and soil microbial community changes and increasing wheat yield. Using the single-factor randomized block trial design, four treatments were employed, the characteristics of greenhouse gas emission, yield and yield components, and the changes of soil microbial community under different compost substitution ratio in the whole wheat growing season were determined by static box-gas chromatography. During the wheat season, both CO_2_ and N_2_O emissions were reduced, whereas CH_4_ emission was increased. That all treatments reduced the Global Warming Potential (GWP) and Greenhouse gas emission intensity (GHGI) in wheat season compared with T0. Compost substitution can alleviate the global warming potential to some extent. Under the condition of compost substitution, the wheat yield under T2 and T3 increased significantly compared with that under the control; however, the spike number and 1000-grain weight did not differ significantly among the treatments. When compost replacement was 30%, the yield was the highest. Under different ratios of compost substitution, the microbial communities mainly comprised Proteobacteria, Actinobacteria, Firmicutes, Patescibacteria, Chloroflexi, Acidobacteria, Bacteroidetes, Gemmatimonadetes, and Verrucomicrobia. The soil microbial community structure differed mainly due to the difference in the compost substitution ratio and was clustered into different groups. In conclusion, to achieve high wheat yield and low greenhouse gas emissions, compost replacement of 30% is the most reasonable means for soil improvement and fertilization.

## 1. Introduction

In China, obtaining high crop yield requires the application of a large amount of chemical fertilizers, leading to an increase in the production cost. Moreover, the long-term application of chemical fertilizers leads to soil acidification and consolidation, and the utilization rate of nitrogen fertilizer is low. Increasing use of chemical fertilizers has deteriorated soil fertility and quality of agricultural products, which has not only affected the comprehensive production capacity of soil but also seriously affected the ecological environment [1]. High-temperature composting of crop straw is one of the effective methods to replace chemical fertilizers [2,3,4]. Straw composting can increase the content of soil organic matter, improve soil physiology and microbial characteristics, promote microbial fixation of nutrients, reduce nutrient loss [5,6], and reduce CH_4_ emissions [7]. However, at the same time, it can lead to an increase in N_2_O emissions [8,9], and promote the recycling of natural resources. Therefore, transformation from inorganic agriculture to ecological and green agriculture is necessary [10]. Use of compost can promote resource utilization of agricultural waste, reduce the use of chemical fertilizers, and improve crop yield and quality, indicating that it has a high practical application value. The combined application of organic and chemical fertilizers is the most efficient approach for improving soil quality, increasing crop yield, and reducing greenhouse gas emissions. The use of chemical fertilizer alone in farmland can lead to an increase in greenhouse gas emissions [11]. Wang et al. [12] reported that the combined application of bio-organic and chemical fertilizers could increase the number of tillers of wheat by 12.4–18.9% and significantly increase the number of effective spikes, grains per spike, and 1000-grain weight of wheat. Other studies have reported that composting can promote plant growth and development and increase crop yield [13]. According to Chaoui et al. [14], composting can increase the number of micro-organisms in soil and improve the availability of soil nutrients. The effect of straw returning on N_2_O emission is uncertain in China and abroad; straw returning has been reported to not only promote N_2_O emission [15], but also inhibit N_2_O emission [16]. Additionally, a few studies reported that using straw compost instead of some part of chemical fertilizer can increase crop yield. The present study investigated the effect of replacing some part of chemical fertilizer with straw compost in terms of differences in growth indices and yield and quality of wheat with different straw compost substitution ratios. The findings may be useful in promoting resource utilization of agricultural waste and reducing the use of chemical fertilizers, thereby providing a theoretical basis for wheat high-yield cultivation techniques.

## 2. Materials and Methods

### 2.1. Experimental Field and Design 

This experiment was conducted in the Anhui University of Science and Technology (E117°33′39″, W32°52′49″) from December 2020 to June 2021, during the field experiment average temperature of 17–8 °C, during the field experiment rainfall of 145 mm. The former stubble of the experiment was rice; the soil was yellow cinnamon soil. The organic matter content in the 0–20-cm soil layer is 20.8 g/kg, with the alkali-hydrolysable nitrogen content of 110.9 mg/kg, available phosphorus content of 25.8 mg/kg, and available potassium content of 115.2 mg/kg.

### 2.2. Test Materials

#### 2.2.1. Production of Compost

The compost used in the experiment comprised cow manure and rice straw harvested after ripening according to the mass ratio of 2:1, and the raw materials were pre-treated before composting. Large chunks of cow dung were simply crushed, and the straw was crushed into small pieces (smaller than 1 cm), which were stacked and set aside. We mixed layers by layers and sprayed water while mixing; the pile was turned repeatedly with a lawn grabber until it was evenly mixed. The overall moisture content was adjusted to 55–65%. The C/N ratio was 25:30, and the oxygen content was 8–18%. The pH was maintained at 6.5–8.0. A small pile with a bottom width of 1.5 m, a height of 1–1.2 m, and a length of 2–3 m was fermented at a high temperature for 50–60 days, and a compost with weak ammonia and manure odor was obtained; the color of rice straw and cow manure changed to granular black–brown after maturity. The nitrogen, P_2_O_5_, K_2_O, and organic matter contents of the compost were 1.03%, 0.87%, 1.35%, and 47.8%, respectively, and the pH was 6.67.

#### 2.2.2. Experimental Wheat

The experimental wheat variety ‘Huaimai 44’ was selected. The seedling stage of this variety is semi-creeping; the leaves are short and green in color. After attaining maturity, the plants become compact, their sword leaf is erect, and the ripening phase is better. The plants exhibit a strong tillering ability, a large number of panicles, earlier yellowing, and strong ability of cold and lodging resistance.

### 2.3. Single Factor Experimental Design

The types of straw compost used as a substitute for chemical fertilizer treatment (T) were as follows: 1. conventional fertilizer control (T0 treatment; compound fertilizer (N18%-P_2_O_5_-18%-K_2_O18%): 600 kg/hm^2^, urea: 300 kg/hm^2^); 2. composting to replace 10% chemical fertilizer (T1 treatment; compound fertilizer (N18%-P_2_O_5_-18%-K_2_O18%): 540 kg/hm^2^, urea: 270 kg/hm^2^, straw compost: 3 t/hm^2^); 3. composting to replace 20% chemical fertilizer (T2 treatment; compound fertilizer (N18%-P_2_O_5_-18%-K_2_O18%): 480 kg/hm^2^, urea: 240 kg/hm^2^, compost: 6 t/hm^2^); and 4. composting to replace 30% chemical fertilizer (T3 treatment; compound fertilizer (N18%-P_2_O_5_-18%-K_2_O18%): 420 kg/hm^2^, urea: 210 kg/hm^2^, compost: 9 t/hm^2^). Each treatment was performed in triplicate. On 5 December 2020, the seeds were sown with an equal row spacing of 15 cm. The sowing rate of 14 m^2^ was 52.50 g/row and that of 15 m^2^ was 56.25 g/row. Except for different fertilization treatments, the other field-management measures adopted the unified management mode of local high-yield wheat field in each district. Wheat was harvested on 9 June 2021.

### 2.4. Observation Indicators and Methods

#### 2.4.1. Collection and Determination of Greenhouse Gases

The closed static chamber method was used to measure the emission fluxes of CH_4_, CO_2_, and N_2_O in wheat field. The sampling device consists of three parts made of opaque organic plastic, namely top box, middle box, and base. In the inner surface of the box (50 cm × 50 cm × 50 cm), a small fan is placed that ensures the uniform distribution of gas. The outer surface consists of a sponge aluminum foil, which is used for reflection and heat insulation, and a thermometer, which is inserted in the upper part of the box and measures the temperature of the box. The upper surface of the base (50 cm × 50 cm × 25 cm) has grooves. During gas production, the grooves are sealed with water, and the box is covered to prevent air leakage between the box and base. The sample was collected through a syringe from the sampling port. The base was buried between the crop rows, exposing only grooves, and it did not move throughout the wheat growing season.

Gas samples were collected during the jointing stage (13 March 2021), booting stage (10 April 2021), flowering stage (24 April 2021), filling stage (9 May 2021), and mature stage (6 May 2021). The sampling time was fixed from 8:00 am to 11:00 am. Water injection in the base tank was closed during gas production; the small fan box at the top of the top box was opened and placed on the base, and the samples were collected every 5 min. Air was extracted from the gas inlet by using a 60 mL syringe at the time intervals of 5, 10, and 15 min, and 60 mL gas was injected into the vacuum air bag for preservation. A total of 3 gas samples were collected. Simultaneously, the temperatures of air and the box were observed and recorded.

The collected samples were analyzed using the Agilent7890B (Agilent, Waldbronn, Germany) gas chromatograph in the laboratory. The analysis column was Porpak.Q packed column. The temperature of the column box was 40 °C, and the carrier gas was high purity N_2_. N_2_O electron capture detector (ECD) was used. The working temperature was 300 °C, and the lowest detection limit was 32 μg·kg^−1^. CO_2_ and CH_4_ were determined using hydrogen detector (FID). The working temperature was 300 °C. The lowest detection limit of CO_2_ and CH_4_ was 4 and 0.2 mg·kg^−1^, respectively. The standard gas of the national standard metrology center was used to calibrate the gas chromatograph, and the external standard working curve was obtained for all 60 samples. The unobserved daily emission fluxes were calculated using the interpolation method; further, the measured values were summed up with the calculated values, and the respective emissions of CO_2_, CH_4_, and N_2_O were obtained. The greenhouse gas emission fluxes were calculated using the following formula:

(1) $$F=\frac{\mathrm{dc}}{\mathrm{dt}}\times \frac{M}{{\;V}_{0}}\times \frac{P}{{\;P}_{0}}\times \frac{{\;T}_{0}}{T}\times H$$

where F is the greenhouse gas emission flux (mg/(m^2^·h)); dc/dt is the slope of the regression curve of the gas volume fraction with time during sampling; M is the molar mass of the gas (g/mol); V_0_ is the molar volume of the gas under the standard gas (22.41 L/mol); P and P_0_ are the air pressure at the sampling point (Pa) and the air pressure at the standard state (101,325 Pa), respectively; T and T_0_ are the absolute temperatures at the time of sampling (K) and in the standard state (273.15 K), respectively; and H is the height of the sampling box (m).

The cumulative greenhouse gas emissions during the wheat growth period were obtained by multiplying the average greenhouse gas emission flux during the two adjacent sampling periods with the time interval between two sampling periods.

#### 2.4.2. Calculation of Comprehensive Warming Potential and Greenhouse Gas Emission Intensity

Using comprehensive warming potential (*GWP*), other greenhouse gas emissions can be converted into equivalent CO_2_, and their climates can be compared [17]. In this study, the *GWP* was used to express the potential effects of different greenhouse gases on global warming. On the 100-year warming scale, the warming potential of CH_4_ and N_2_O is 28 and 265 times of that of CO_2_. The *GWP* can be calculated from the cumulative emission of each gas and its corresponding temperature increasing coefficient.

*GWP* = CO_2_ + 25 × CH_4_ + 298 × N_2_O (2)

*GHGI* was used to evaluate the comprehensive greenhouse effect of each treatment. The algorithm of *GHGI* is:

*GHHI* = *GWP*/*Y* (3)

where *GHGI* is the greenhouse gas emission intensity of the treatment, and *Y* is the crop yield of each treatment (kg·hm^−2^).

#### 2.4.3. Determination of Yield

Representative 1 m, 2-row samples were selected from each plot during the wheat maturity stage. The average spike number was investigated, and the spike number per unit area was calculated. Overall, 20 plants were selected from each plot for indoor seed testing, and the average grain number per spike and 1000-grain weight were investigated.

#### 2.4.4. Soil sample Collection and Treatment

Soil samples were collected in June 2021 (after wheat harvest). After removing the surface floating soil, according to the ‘Z’-shaped sampling route, 0–20 cm soil cores were randomly selected using a sampler (diameter 2.5 cm). Then, the samples were mixed evenly to remove impurities such as gravel and plant residual roots. The fresh soil samples were divided into two parts: one part was air-dried for the determination of soil physical and chemical properties, and the other part was preserved at −80 °C for the extraction of soil genomic DNA.

#### 2.4.5. Extraction and Sequencing of Soil DNA

In total, 0.5 g of soil stored in a refrigerator at −80 °C was taken, and total DNA of the soil was extracted using the PowerSoil^®^ DNA extraction kit (MoBio Laboratories, Inc., Carlsbad, CA, USA), according to the manufacturer’s instructions. DNA content was quantified using a NanoDrop spectrophotometer (ND-2000, Thermo Scientific, Waltham, MA, USA), diluted to 10 ng·μ L^−1^, and stored in a refrigerator at −20 °C util use. Each DNA sample had 9 PCR repeats and included 2 negative controls without DNA template. The PCR reaction system and reaction procedure were based on the method described by Zhao et al. [18]. The PCR product was purified through gel cutting, and the concentration was determined using PicoGreen^®^ (Promega, Madison, WI, USA). After equimolar mixing, the product was sent to Guangdong MAGIGENE Technology limited company for sequencing.

#### 2.4.6. Statistical Analysis

The test data were processed and analyzed using MS Excel 2010 and DPS7.05 software, and the significance was tested using the LSD method. The relationship between soil physical and chemical properties and greenhouse gases and their functional bacteria was analyzed using the ‘psych’ package in R, and the principal coordinate analysis (pCoA) and redundancy analysis (RDA) of community structure differences and environmental factors on community structure were completed using ‘vegan’ in R. The ‘pheatmap’ and ‘ggplot2’ packages in Excel and R were used for drawing.

## 3. Results

### 3.1. Effects of Different Compost Substitution Ratios on Soil N_2_O, CH_4_, and CO_2_ Emissions

**Figure 1:**
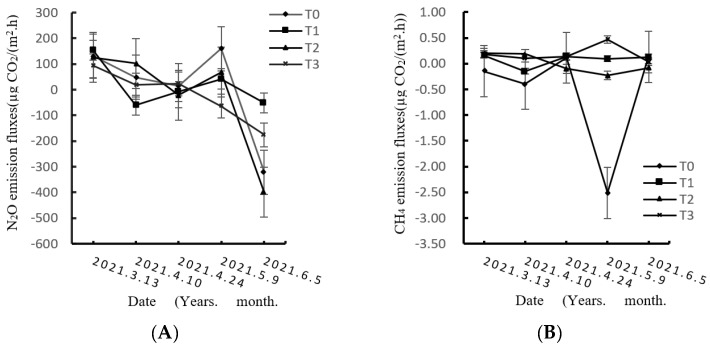

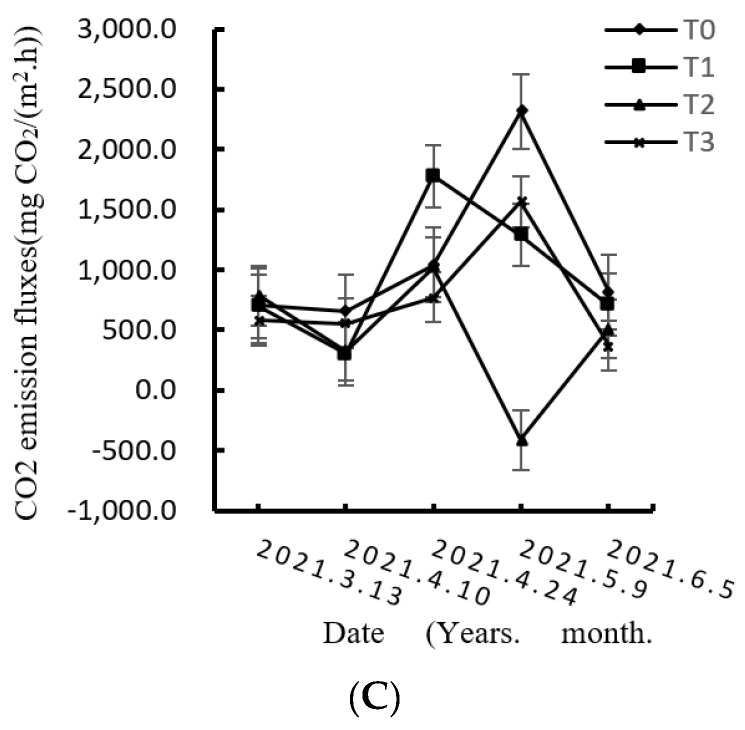
Dynamics of (**A**) N_2_O flux, (**B**) CH4 flux, (**C**) CO_2_ flux under different treatments.

Figure 1 shows the dynamic changes in N_2_O, CH_4_, and CO_2_ emission fluxes during the wheat growth period under different compost substitution ratios. As shown in Figure 1, under different compost substitution ratios, the flux emission of each treatment displayed a unimodal pattern, and the emission flux demonstrated first an increasing trend and then a decreasing trend with the seasonal change; the maximum emission flux was obtained at the jointing stage after sowing. The emission of N_2_O after T1 treatment was higher than that after T0 treatment, and the amount of N_2_O uptake after T2 treatment was higher than that after T3 treatment.

The flux of CH_4_ was both positive and negative, and it fluctuated rapidly. As shown in Table 1, during the wheat growth period, the cumulative emission of CH_4_ in each treatment was negative, suggesting absorption. The amount of absorption under T0 treatment was higher than that under T2 treatment, and there was a positive value. The emission under T3 treatment was higher than that under T1 treatment. Outcomes under each treatment differed significantly from those under T0 (*p* < 0.05).

The emission flux of CO_2_ from each treatment clearly changed during the growth period of wheat, showing a single peak. The emission flux of CO_2_ increased gradually after low performance at the booting stage, reached the peak at the filling stage, and then decreased. According to Table 1, the cumulative CO_2_ emission of each treatment was in the order T0 > T1 > T3 > T2 during the wheat growth period. The cumulative emission of 55,731.98 kgC·hm^−2^ under T1, T2, and T3 decreased significantly by 13.6%, 59.7%, and 30.5%, respectively, compared with that under T0 treatment (*p* < 0.05). Outcomes under each treatment differed significantly from those under T0 (*p* < 0.05).

### 3.2. GWP and GHGI under Different Compost Substitution Ratios

The estimated results of GWP and GHGI under different compost substitution ratios during the wheat growth period are presented in Table 1. T0 treatment made the greatest contribution to the comprehensive warming potential of farmland. The comprehensive warming potential of TI, T2, and T3 decreased by 13.8%, 149.8%, and 42.6%, respectively, compared with that of T0. With significant variations in the yield under each treatment, both GHGI and GWP exhibited a different trend. Under different compost substitution ratios, the GHGI under different treatments was in the order T1 > T3 > T2 > T0. The GHGI under T1 was the highest (42.53 g·CO_2_-eq kg^−1^), although T1 exhibited the lowest yield among all treatments.

### 3.3. Effects of Different Compost Substitution Ratios on Wheat Yield and Yield Components

The effects of different compost substitution ratios on wheat yield, quality, and yield components are shown in Table 2. The number of grains per spike in T2 was significantly higher than those in T0, T1, and T3; however, the difference in the number of grains per spike between T0 and T3 was nonsignificant. The number of panicles in T1 was significantly lower than those in T0, T2, and T3. In terms of the 1000-grain weight, T0, T1, and T2 exhibited no significant difference; however, 1000-grain weight after T3 was significantly higher than that after other treatments. Overall, 30% substitution of compost significantly increased the wheat yield, and the yield after T3 (9647.01 kg·hm^−2^) increased by 21.3% compared with that under T0. The yield under T0 and T2 was significantly higher than that under T1; however, it did not differ significantly between T0 and T2.

### 3.4. Effect of Microbial Community Composition under Different Compost Substitution Ratios

**Figure 2:**
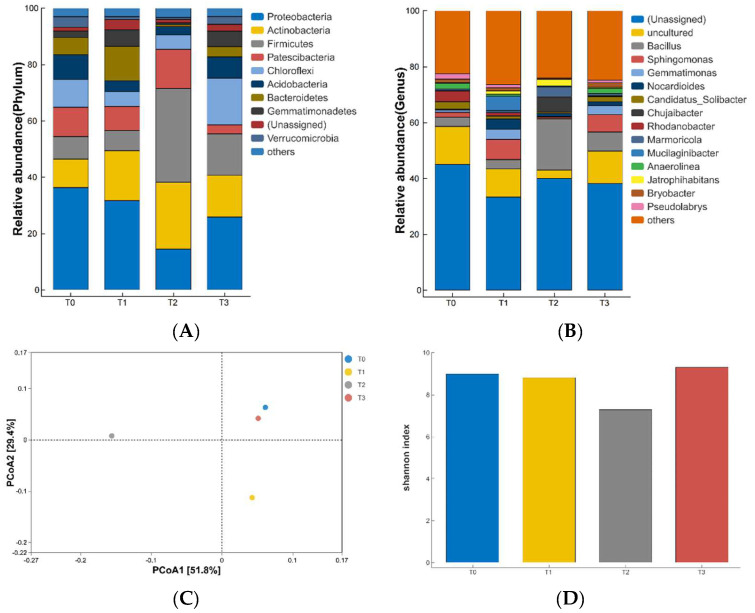
The relative abundance (**A**), the relative abundance of dominant functional bacteria (>1%) (**B**), the PCoA (**C**), and fragrance index (**D**) of the differences in community structure under different compost substitution ratios.

Figure 2A shows the relative abundance of microbial community structure under different compost substitution ratios. The microbial community under different treatments comprised mainly Proteobacteria, Actinobacteria, Firmicutes, Patescibacteria, Chloroflexi, Acidobacteria, Bacteroidetes, Gemmatimonadetes, and Verrucomicrobia. With an increase in the compost substitution ratio, the relative abundance of Actinomycetes under T0 increased significantly compared with those under T1, T2, and T3 (*p* < 0.05), but it decreased when the substitution ratio increased to 30%. The relative abundance of Proteus was significantly lower under T2 than those under T0, T1, and T3 (*p* < 0.01). The relative abundance of thick-walled bacteria was significantly higher under T2 than those under T0, T1, and T3 (*p* < 0.01). The relative abundance of Patescibacteria was significantly lower under T3 than those under T0, T1, and T2 (*p* < 0.01). The relative abundance of Campylobacter was significantly higher under T0 and T3 than that under T1 and T2 (*p* < 0.01). Likewise, the relative abundance of acid bacilli was significantly lower under T1 and T2 than that under T0 and T3 (*p* < 0.01).

Figure 2B shows the relative abundance of microbial communities under different compost substitution ratios at the genus level, including 13 dominant functional bacteria (relative abundance more than 1%) and various α-, β-, and γ-type amoeba (Proteobacteria). The relative abundance of *Bacillus* was significantly higher under T2 than those in T0, T1, and T3. For *Sphingomonas*, the relative abundance was significantly higher under T1 and T3 than that under T0 and T2. The relative abundance of *Gemmatimonas* was significantly lower under T0 and T2 than that under T1 and T3. The relative abundance of *Nocardioides* was significantly higher under T1 than that under T0, T2, and T3 (*p* < 0.01).

Figure 2C presents the OTU data based on 97% similarity, and the weighted (Weighted Unifrac) algorithm was used to stack the results of pCoA of microbial community structure under different compost substitution ratios. Among them, the variance contribution rates of the first and second principal components were 51.8% and 29.4%, respectively, and the cumulative variance contribution rate was 81.2%. On the first axis, T1 and T2 could be well separated on the first axis, and the amount of variance explained was 51.8%.

Figure 2D shows the results of Shannon index diversity analysis of the microbial community structure. The higher the Shannon index, the richer is the microbial community diversity, and vice-a-versa. The Shannon index under T2 was significantly lower than that under other three treatments (*p* < 0.05); however, no significant difference was observed in terms of diversity among the other three treatments.

### 3.5. Redundancy Analysis of the Microbial Community Structure, Environmental Factors, and Soil Nutrient Content

**Figure 3:**
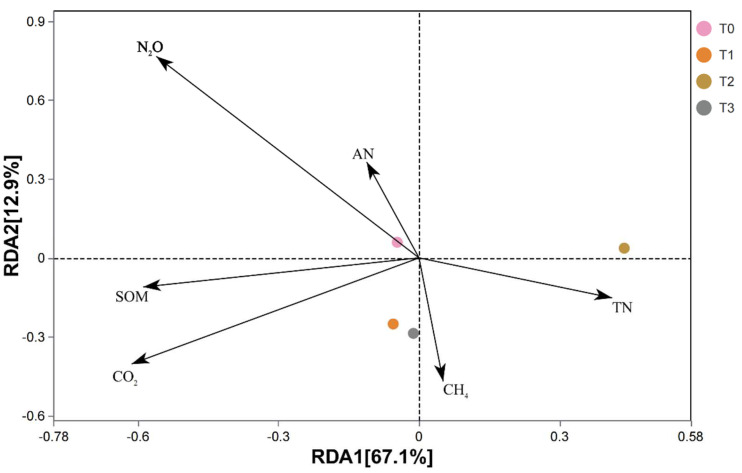
Redundancy analysis of environmental factors, soil nutrient content, and soil microbial community structure.

The correlation between the microbial community structure, environmental factors, and soil nutrient content was further examined, and the results are shown in Figure 3. A total of 80% of the relationship between microbial community structure, environmental factors, and soil nutrient content could be explained by the two axes, reflecting the effects of environmental factors and soil nutrient content on the soil microbial community structure. Further analysis indicated that the amount of variance explained by RDA1 and RDA2 was 67.1% and 12.9%, respectively, and the responses of soil microbial communities to environmental factors and soil nutrients were different under different compost treatments. Among them, T0, T1, and T3 positively correlated with AN, N_2_O, SOM, and CO_2_, whereas T2 positively correlated with TN and CH_4_.

## 4. Discussion

Many studies have reported that the application of compost can improve the physical and chemical properties of soil [19,20,21,22], increase the content of soil nutrients [19,20,23,24], and increase the diversity and abundance of soil micro-organisms [14,25,26,27]. Moreover, compost application is beneficial to the transformation and release of soil nutrients [14]. Greenhouse gas emissions from farmland are affected by many factors such as climatic conditions, soil characteristics, fertilizer types, and agricultural management measures [27,28,29,30].

N_2_O is emitted from soil by the production of ammonium salts during the process of oxidation involving nitrifying bacteria [31]. Some studies have suggested that compost substitution can change the soil characteristics [32], and stimulate the soil microbial activity to increase soil microbial biomass, thereby promoting denitrification to increase N_2_O emission [33,34,35]. A study by Guo et al. [36] indicated that compost substitution can significantly reduce soil N_2_O emission, consistent with the findings of the present study.

The emission flux of N_2_O is relatively high at the jointing stage, which may be related to fertilization. Fertilization can provide a large amount of available nitrogen to soil micro-organisms and accelerate the processes of microbial nitrification and denitrification, thus promoting N_2_O emission [37]. Under the compost substitution ratio of 20%, the cumulative N_2_O emission was the lowest, which may be attributed to the low Shannon index and poor microbial community diversity. Studies have reported that micro-organisms, particularly Proteus, thick-walled bacteria, and Actinomycetes, are involved in straw decomposition [38]. Nitrogen-fixing micro-organisms mainly include various α-, β-, and γ-type Proteobacteria [39]. Among these groups, Firmicutes can participate in straw decomposition and are the most abundant bacteria that play a leading role in nitrogen utilization and compete with nitrifying bacteria for nitrogen sources; thus, they promote the fixation of plant available nitrogen, reduce the substrate for N_2_O production [40,41], and inhibit N_2_O emissions.

Previous studies have reported the involvement of micro-organisms in various carbon cycle metabolic processes such as the conversion of inorganic carbon to organic carbon, production of methane and methane oxidation, and decomposition of organic matter [42]. Some studies have reported that the amount of CH_4_ emitted from dryland soil is relatively low, which is mostly shown as absorption. This may be due to soil dryness, good aeration conditions, and abundant oxygen in the soil, which make CH_4_ easy to be oxidized [43]. It may also be due to the rapid decomposition of organic matter and slow accumulation of organic carbon in dryland soil, thus affecting the production and emission of CH_4_ [44]. In the present study, the cumulative emission of CH_4_ increased under different compost substitution ratios, and the soil emission of CH_4_ was increased by the compost substitution treatment. This may be attributed to the fact that the relative abundance of *Bacillus*, Actinobacteria, and *Sphingomonas* increased after compost substitution, which reduced the decomposition of soil organic matter and promoted its rapid accumulation, thereby increasing the production and emission of CH_4_. The absorption under T0 was higher than that under T1, T2, and T3, which may be due to the presence of fewer *Proteus* in the soil without composting and increased fixation of organic matter in the soil [39], leading to a decrease in the methanogen matrix and CH_4_ emission.

In this study, CO_2_ emissions reduced under different compost substitution ratios, and the relative abundance of *Bacillus* increased after compost substitution. This resulted in soil carbon sequestration, leading to a decrease in the decomposition of organic matter by micro-organisms and the transformation of mineral nutrients. Different compost substitution ratios lead to differences in the amount of soil organic carbon fixed. The stability of soil organic carbon increases gradually, and the deep organic carbon is not easy to be used by biology. Therefore, soil respiration is relatively weak, and CO_2_ emissions decrease.

In this study, all treatments reduced the comprehensive warming potential of greenhouse gases in the wheat season, and the comprehensive warming potential under T3 treatment was relatively low, which may be due to the lack of carbon-fixing micro-organisms in T3, rendering the soil to fix a limited amount of organic carbon. The comprehensive warming potential under T2 decreased by 60% compared with that under T0, which indicated that soil organic carbon was fixed due to compost substitution. The GHGI under T2 was the weakest among all compost substitution treatments, indicating that compost substitution could not only reduce the GHGI but also enhance the carbon sequestration of soil micro-organisms and improve soil productivity.

Additionally, compost substitution increased wheat yield, which is consistent with the findings of a study by Wang Jiabao [12]. The yield increased the most under compost replacement of 30%. The compost replacement of 30% increased the abundance of micro-organisms causing soil carbon sequestration and improved soil productivity, in addition to significantly increasing the effective panicles and 1000-grain weight of wheat. Thus, compost substitution is an ideal cultivation measure for soil improvement and fertilization.

## 5. Conclusions

Throughout the wheat season, compost substitution significantly reduced CO_2_ and N_2_O emissions, and the accumulation of CO_2_ under all treatments was lower than that under T0. 20% of compost substitution was the smallest and the largest without composting. N_2_O accumulation was also the lowest under compost replacement of 20%; whereas for CH_4_, it was emission. In terms of GWP and GHGI, compost substitution of 30% could reduce GHGI and significantly increase wheat yield by 21.3% on a 100-year scale. Therefore, compost replacement of 30% could relatively reduce greenhouse gas emissions and significantly increase wheat production, indicating that compost replacement of 30% could be beneficial to both the economy and environment.

## Figures and Tables

**Table 1:** Accumulation of greenhouse gas emissions, comprehensive greenhouse gas warming potential, and greenhouse gas emission intensity under different compost substitution ratios.

Treatment	N_2_O Cumulative Emission (kg N∙hm^−2^)	CH_4_ Cumulative Emission (kgC∙hm^−2^)	CO_2_ Cumulative Emission (kg C∙hm^−2^)	GWP (kgCO_2_-eq·hm^−2^)	GHGI (g CO_2_-eq·kg^−1^)
T0	0.32 ± 0.01 b	−28.21 ± 0.71 d	55,731.98 ± 1393.30 a	55,121.75 a	6.93 a
T1	0.74 ± 0.02 a	3.74 ± 0.09 b	48,128.64 ± 1203.22 b	48,442.61 b	6.56 a
T2	−1.26 ± 0.03 c	−0.22 ± 0.01 c	22,451.49 ± 561.29 d	22,070.57 d	2.63 c
T3	−1.07 ± 0.03 c	9.39 ± 0.23 a	38,729.71 ± 968.24 c	38,645.56 c	4.01 b

Note: Values followed by different lowercase letters in a column indicate significant difference among treatments at the 0.05 level.

**Table 2:** Effects of different compost substitution ratios on wheat yield and yield components.

Treatments	Spike Number (Ear/each)	Kernels Per Spike (Per Spike)	1000-Grainweight (g)	Yield (kg/hm^2^)
T0	620.00 ± 2.65 a	32.38 ± 1.00 c	39.61 ± 4.03 b	7950.35 ± 33.93 b
T1	566.00 ± 4.93 b	35.90 ± 0.29 b	36.32 ± 1.61 b	7380.70 ± 64.32 c
T2	624.67 ± 16.34 a	38.92 ± 0.67 a	34.41 ± 2.55 b	8363.91 ± 218.83 b
T3	630.67 ± 11.84 a	31.18 ± 0.74 c	49.07 ± 0.59 a	9647.01 ± 181.06 a

Note: Values followed by different lowercase letters in a column indicate significant difference among treatments at the 0.05 level.

## Data Availability

Not applicable.
